# PML Nuclear Bodies and SATB1 Are Associated with HLA Class I Expression in EBV+ Hodgkin Lymphoma

**DOI:** 10.1371/journal.pone.0072930

**Published:** 2013-08-29

**Authors:** Yuxuan Liu, Anke van den Berg, Rianne Veenstra, Bea Rutgers, Ilja Nolte, Gustaaf van Imhoff, Lydia Visser, Arjan Diepstra

**Affiliations:** 1 Department of Pathology & Medical Biology, University of Groningen, University Medical Center Groningen, Groningen, The Netherlands; 2 Department of Epidemiology, University of Groningen, University Medical Center Groningen, Groningen, The Netherlands; 3 Department of Hematology, University of Groningen, University Medical Center Groningen, Groningen, The Netherlands; University of Birmingham, United Kingdom

## Abstract

Tumor cells of classical Hodgkin lymphoma (cHL) are characterized by a general loss of B cell phenotype, whereas antigen presenting properties are commonly retained. HLA class I is expressed in most EBV+ cHL cases, with an even enhanced expression in a proportion of the cases. Promyelocytic leukemia protein (PML) and special AT-rich region binding protein 1 (SATB1) are two global chromatin organizing proteins that have been shown to regulate HLA class I expression in Jurkat cells. We analyzed HLA class I, number of PML nuclear bodies (NBs) and SATB1 expression in tumor cells of 54 EBV+ cHL cases and used 27 EBV− cHL cases as controls. There was a significant difference in presence of HLA class I staining between EBV+ and EBV− cases (p<0.0001). We observed normal HLA class I expression in 35% of the EBV+ and in 19% of the EBV− cases. A stronger than normal HLA class I expression was observed in approximately 40% of EBV+ cHL and not in EBV− cHL cases. 36 EBV+ cHL cases contained less than 10 PML-NBs per tumor cell, whereas 16 cases contained more than 10 PML-NBs. The number of PML-NBs was positively correlated to the level of HLA class I expression (p<0.01). The percentage of SATB1 positive cells varied between 0% to 100% in tumor cells and was inversely correlated with the level of HLA class I expression, but only between normal and strong expression (p<0.05). Multivariable analysis indicated that the number of PML-NBs and the percentage of SATB1+ tumor cells are independent factors affecting HLA class I expression in EBV+ cHL. In conclusion, both PML and SATB1 are correlated to HLA class I expression levels in EBV+ cHL.

## Introduction

Classical Hodgkin lymphoma (cHL) is characterized by a minority of B cell derived tumor cells, named Hodgkin Reed-Sternberg cells (HRS cells) and an abundant reactive infiltrate. Despite the loss of B cell phenotype [1], HRS cells often retain their professional antigen presenting phenotype, including expression of human leukocyte antigens (HLA). They also express transporters of antigenic peptides (TAP1, TAP2), CD74, and tapasin required for proper antigen presentation, as well as co-stimulatory (B7, CD40, CD70) and cell adhesion molecules (ICAM-1 and LFA-3) [2].

Epstein Barr virus (EBV) is present in the tumor cells in approximately 30% of the cHL patients in Western Europe and plays a causal role in cHL pathogenesis [3]. The human leukocyte antigen (HLA) is a crucial component of the human immune system as it presents antigens to T cells. HLA class I restricted CD8+ cytotoxic T-cell (CTL) responses are known to target EBV infected cells through cell-mediated immunity [4]. HLA class I expression is retained in HRS cells in ∼70% of the EBV+ cHL cases [5]. This is remarkable, since HLA class I restricted immune responses to EBV derived antigenic peptides should be detrimental to EBV+ HRS cells. Previous observations showed an enhanced HLA class I staining intensity in a proportion of the EBV+ cHL cases as compared to the surrounding lymphocytes [6], [7]. In contrast, only ∼15% of the EBV− cases retain HLA class I expression and positive cases showed a staining intensity similar to lymphocytes in the reactive infiltrate [5].

Promyelocytic leukemia protein (PML) and special AT-rich region binding protein 1 (SATB1) are two proteins that have been shown to regulate HLA class I expression. PML is the main component of nuclear bodies (NBs). PML-NBs are discrete nuclear foci with a diameter of 0.2 to 1.0 µm. They are present in the nuclei of most mammalian cells. PML-NBs are dynamic nuclear matrix-associated domains that recruit a variety of proteins and regulate many nuclear functions such as DNA replication, transcription and epigenetic modifications (reviewed in [8]). There are 5 to 30 PML-NBs per nucleus depending on the cell type, cell cycle phase and differentiation stage [9]. PML can be processed into seven different transcripts resulting in protein isoforms that have variable C-termini [10]. PML-NBs associate with the HLA locus and other transcriptionally active genomic regions [11], [12]. PML induces the expression of HLA class I heavy chains and β2-microglobulin at the level of transcription, thereby restoring defective antigen presentation in lung cancer cell lines [13]. Down regulation of PML results in a reduced HLA-A (classical antigen presenting HLA class I) but an enhanced HLA-G (non-classical, non-antigen presenting HLA class I) expression in Jurkat cells [14]. SATB1 is highly abundant in T cells and acts as a global chromatin organizer. It is required for the assembly and maintenance of a higher-order chromatin loop architecture and it consequently regulates gene expression [15], [16], [17]. SATB1 partially resides within PML-NBs and has been shown to organize the HLA class I locus into distinct loops. Silencing of SATB1 in Jurkat cells results in an enhanced expression of HLA-A, HLA-G, HLA-H and HCG4P6 [14].

The mechanism of the altered HLA class I expression levels (stronger than normal or lost) observed in HRS cells in part of the EBV+ cHL cases is currently unknown. In this study, we investigated a possible association between PML-NBs, SATB1 and HLA class I expression in the HRS cells of EBV+ cHL.

## Materials and Methods

### Patients

We selected 54 primary EBV+ cHL cases and as a control 27 random primary EBV− cHL cases [18]. All cases were diagnosed between 1987 and 2009. The age at diagnosis ranged from 7 to 94 years. Tissues were retrieved from the tissue bank of the Pathology Department, University Medical Center Groningen. All diagnoses were established according to the classification guidelines of the World Health Organization [19]. The protocol was approved by the medical ethics board of the University Medical Center Groningen [18]. All protocols were carried out in accordance with the Declaration of Helsinki.

### Immunohistochemical Staining and Scoring of HLA Class I, PML-NBs and SATB1

4-µm thick paraffin sections were deparaffinized by xylene and rehydrated through a graded ethanol series. Tissue sections were incubated with 0.3% hydrogen peroxide for 30 min to block endogenous peroxidase and heated in 1 mM Tris/EDTA solution (pH 9.0) for antigen retrieval. The slides were washed with PBS, and incubated with the primary antibodies against HLA class I, ß2-microglobulin, PML and SATB1 (Table S1). The PML antibody detects all PML isoforms. The surrounding infiltrate served as a positive internal control, while PBS without primary antibody served as a negative control. Positive staining was visualized with 3,3-diaminobenzidine (DAB) after incubation with secondary and tertiary antibodies. Sections were counterstained with hematoxylin.

HLA class I heavy chain staining was scored for each case side by side with ß2-microglobulin staining. The surrounding inflammatory cells were used as a reference for assessing the staining intensity. The staining intensity was scored as normal, i.e. similar to reactive lymphocytes, or strong, i.e. stronger than the reactive lymphocytes. Cases without membranous staining were scored as negative. For PML, we scored HRS cells based on the average number of PML-NBs in the nuclei of at least 20 HRS cells. The number of small discrete foci in the nucleus of HRS cells was counted. Initially, four scoring categories were used (average number of PML-NBs <6, 6–10, 11–15 or >15). Because the number of cases in the <6 and >15 categories were quite small, cases were grouped into two categories; with 10 or less PML-NBs per cell or >10 PML-NBs per cell (per 4 µm tissue section). In three random cases with <5 PML-NBs per nucleus and three random cases with >15 PML-NBs per nucleus we measured the nuclear surface area of 20 individual tumor cells in each case (120 tumor cells in total) by Aperio ImageScope software v11.1 after digitally capturing the slides (Aperio Scanscope). For SATB1, we scored the nuclear staining intensity and the percentage of SATB1 positive HRS cells. If there was no nuclear signal in tumor cells, we defined them as negative. If HRS cells stained weaker than the surrounding lymphocytes, we defined them as weakly positive. If HRS cells and surrounding cells had the same or a (slightly) stronger intensity as the surrounding infiltrating cells, we defined them as positive.

### Statistical Analysis

Differences between HLA class I expression defined subgroups were evaluated by the Chi-square test or Fisher exact test. The Kruskal-Wallis test and post-hoc Dunn’s multiple comparison tests were used to test for statistically significant differences in age distribution and percentages of SATB1 positive HRS cells.For SATB1 staining intensity negative and weakly positive scores were grouped together. Linear regression analysis was used to evaluate whether the number of PML-NBs per nucleus was correlated to the nuclear surface area. Ordinal regression was used for multivariable analysis of factors contributing to HLA class I expression (factors with a p-value <0.1 in univariable analysis were included). P values less than 0.05 were considered to be significant. SPSS 20.0 was used for the analyses.

## Results

### HLA Class I Expression and Patient Characteristics in Relation to EBV Status

**Figure 1:**
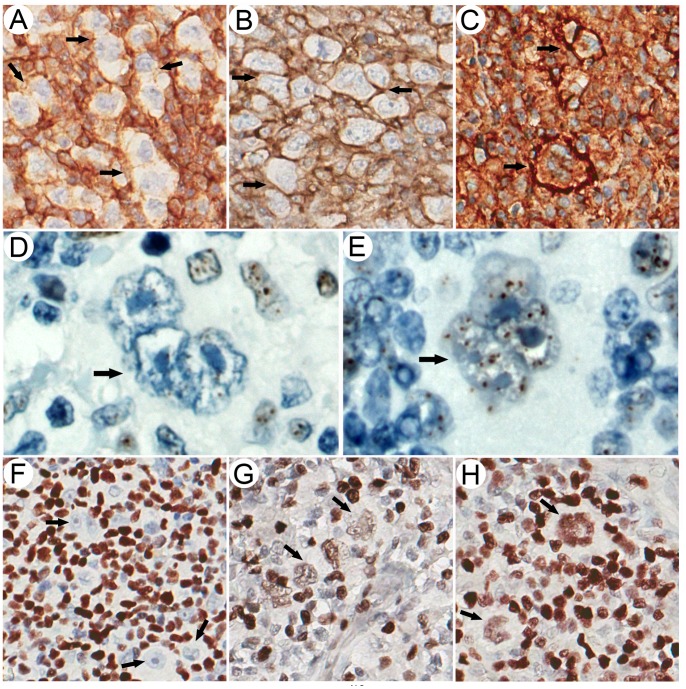
Representative immunostaining results of HLA class I, SATB1 and PML-NBs. Arrows indicate the HRS cells. (**A**) HLA class I negative case. There is no membranous staining between adjacent HRS cells, while membranes of surrounding cells were positive. (**B**) HLA class I positive case. Several adjacent HRS cells show positive membranous staining at the same intensity as the surrounding lymphocytes. (**C**) cHL case with a strongly positive HLA class I staining. Staining intensity of HRS cells is more pronounced than the intensity of the surrounding lymphocytes. (**D**) cHL case with an average of ≤10 PML-NBs per cell. One HRS cells with 4 tiny PML-NBs in the nucleus is shown. (**E**) cHL case with an average of >10 PML-NBs per cell. A representative HRS cell with more than 20 PML-NBs in the nucleus is shown. (**F**) cHL case without nuclear SATB1 staining in HRS cells. (**G**) Weakly positive nuclear SATB1 staining in HRS cells. (**H**) Positive nuclear SATB1 staining in HRS cells.

EBV+ tumor cell status was associated with mixed cellularity subtype and age (Table S2). Representative cases with negative, normal and strongly positive HLA class I staining in HRS cells are shown in Fig. 1A–C. HLA class I staining results were consistent with the ß2-microglobulin staining results in all cases (data not shown). In the EBV− group, none of the cases showed a strong positive staining, 5 cases showed a normal staining intensity and 22 cases were negative for HLA class I. In the EBV+ group, 21 cases stained strongly positive, 19 showed a normal intensity and 14 cases were negative for HLA class I. This difference in HLA class I staining pattern was significantly different (P<0.0001; Table S2). Age, gender and clinical stage had no correlation with tumor cell HLA class I staining intensity in the EBV+ cHL patients. Significant differences were observed in the proportion of HLA class I negative, normal and strong positive cases between histological subtypes (P = 0.03, Table 1). All negative cases were of the nodular sclerosis subtype.

**Table 1:** Statistical analysis of the correlation of clinicopathological characteristics, SATB1 and PML-NBs with HLA class I staining patterns in EBV+ cHL (n = 54).

	HLA class I staining						Univariableanalysis	Multivariable analysis
	Negative		normal		strong		*p* value	*p* value^d^
	n	%	n	%	n	%		
**Age, median**								
median	22		49		56		0.094^a^	0.116
(min-max)	(8–72)		(8–88)		(7–94)			
**Age, year**								
0–14	3	33.3	3	33.3	3	33.3		
15–44	7	41.2	5	29.4	5	29.4	N.S.^b^	–
45–64	3	18.8	6	37.5	7	43.8		
> = 65	1	8.3	5	41.7	6	50.0		
**Gender**								
M	6	18.2	12	36.4	15	45.4	N.S.^b^	–
F	8	38.1	7	33.3	6	28.6		
**Clinical stage**								
I	1	7.1	5	35.7	8	57.1		
II	7	41.2	5	29.4	5	29.4	N.S.^c^	–
III	6	46.2	3	23.1	4	30.8		
IV	0	0.0	1	100.0	0	0.0		
unknown	0	0.0	5	55.6	4	44.4		
**Histological subtype**								
Nodular Sclerosis	14	40.0	8	22.9	13	37.1	0.026^c^	0.093
Mixed Cellularity	0	0.0	5	50.0	5	50.0		
LR/LD/NOS	0	0.0	6	66.7	3	33.3		
**Number of PML-NBs**								
0–10 PML-NBs	13	34.2	16	42.1	9	23.7	0.002^b^	0.008
>10 PML-NBs	1	6.3	3	18.8	12	75.0		
**% of SATB1+ HRS cells** e								
Median	65		100		40		0.046^a^	0.024
(min-max)	(0–100)		(0–100)		(0–100)			
**SATB1 staining intensity**								
negative/weakly positive	8	33.3	4	16.7	12	50.0	0.039^b^	–
positive	6	20.0	15	50.0	9	30.0		

a Kruskal-Wallis test;

b Chi-square test;

c Fisher’s exact test;

d Ordinal regression analysis for factors with a p value <0.1 in univariable analysis;

e SATB1 staining intensity was left out of the model since it correlates to % of SATB1+ HRS cells. N.S., not significant. LR, lymphocyte rich. LD, lymphocyte deleted. NOS, not otherwise specified.

### PML Staining in Relation to HLA Class I Expression in EBV+ cHL

**Figure 2:**
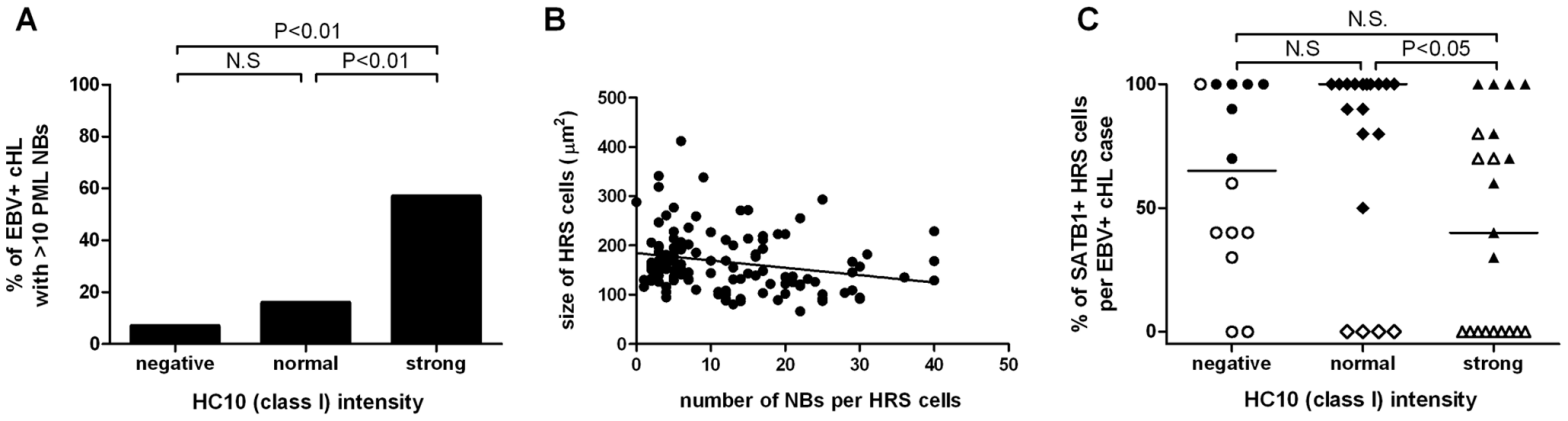
PML and SATB1 staining results in relation to HLA class I in EBV+ cHL (n = 54). (A) The percentage of EBV+ cHL with >10 PML-NBs significantly increased from HLA class I negative to normal and strong positive cHL cases (p = 0.002, Chi-square). Post-hoc test indicated that the percentage of EBV+ cHL cases with >10 PML-NBs differs significantly between HLA class I negative and strong positive or normal and strong positive cHL cases. (**B**) Correlation of the number of PML-NBs with nuclear size in 120 HRS cells from 6 different cases (three with low number and three with a high number of PML-NBs). There is a weak negative correlation between the number of PML-NBs and size of the tumor cell nucleus (P = 0.01). (**C**) The percentage of SATB1 positive HRS cells in EBV+ cHL cases with negative, normal or strong HLA class I staining pattern differ significantly (p = 0.046, Kruskal-Wallis test). Dunn’s multiple comparison test indicated a significant difference specifically between the HLA class I normal and strong staining groups. The median SATB1 percentage is indicated in each group. White symbols indicate the cases with no or weakly positive SATB1 staining, whereas black symbols indicate cases with positive SATB1 staining.

The PML antibody stained PML-NBs as randomly distributed discrete nuclear dots in virtually all nuclei in the tissue. There was no diffuse nuclear PML staining. To correlate HLA class I to PML, a known regulator of HLA class I overexpression, the average number of PML-NBs per HRS cell was scored in EBV+ cHL (Table S3). Representative cases are shown in Fig. 1D and 1E. 38 cHL cases had an average of ≤10 PML-NBs per cell, while 16 cases showed an average of >10 PML-NBs per cell. The percentage of EBV+ cHL cases with >10 PML-NBs was significantly different between HLA class I groups, with the lowest frequency in HLA class I negative cases (1 out of 14, i.e. 7%), an intermediate frequency in HLA class I normal (3 out of 19, i.e. 16%) and the highest percentage in HLA class I strong positive (12 out of 21, i.e. 57%) cHL cases (p = 0.002, Fig. 2A). Post-hoc test indicated that the percentage of EBV+ cHL cases with >10 PML-NBs significantly differed between HLA class I negative and strong positive (P = 0.004) and between normal and strong positive cHL cases (P = 0.01). Strikingly, the 7 cases with the highest number of PML-NBs per HRS cell all had a strongly positive HLA class I staining. Thus, a high number of PML-NBs in HRS cells is correlated with enhanced HLA class I expression in EBV+ cHL.

To explore if the number of PML-NBs counted in a 4 µm tissue section is related to the size of the nucleus, we correlated the number of PML-NBs to the nuclear surface area in 20 individual tumor cells per case in 6 different cases (3 cases with low PML-NB numbers and 3 with high PML-NB numbers). The surface area of HRS cell nuclei in 4 µm tissue sections ranged from 66 to 412 µm^2^ and the number of PML-NBs per nucleus ranged from 0 to 40. A weak negative correlation was observed with bigger nuclei having slightly less PML-NBs (p = 0.01, r^2^ = 0.05, Figure 2B). Considering HRS cell nuclei as a sphere, we estimate that there are close to zero to up to 170 PML-NBs in a complete HRS cell nucleus. In contrast, we saw on average only 3 to 5 PML-NBs per lymphocyte nucleus in the reactive infiltrate.

### SATB1 Staining in Relation to HLA Class I Expression in EBV+ cHL

The percentage of SATB1 positive HRS cells varied from 0–100% in EBV+ cHL. Representative cHL cases with negative, weakly positive and positive SATB1 nuclear staining are shown in Fig. 1F–H. Weak SATB1 staining coincided with a highly variable percentage of SATB1 positive HRS cells, while positive SATB1 staining was related to higher percentages of SATB1 positive HRS cells (usually >50%). HLA class I results were compared to both the percentage and staining intensity of SATB1+ HRS cells. We observed a significant difference in the percentages of SATB1 positive cells between the HLA class I negative, normal and strongly positive groups (median 65%, 100% and 40%, p = 0.046, Fig. 2C) and post-hoc analysis showed that this was caused by the difference between normal and strong HLA class I staining. In the EBV+ group, 30 cases stained positive, whereas 24 cases were negative or showed a weak staining for SATB1. There was a significant difference of SATB1 staining intensity among HLA class I negative, normal and strong positive groups (P = 0.039, Table 1). Post-hoc analysis indicated that this effect is due to a significant difference between HLA class I normal and strong positive groups, the latter being associated with negative/weakly positive SATB1 staining. There was no statistical difference between HLA class I negative vs. normal or negative vs. strong cases.

### Multivariable Analysis of Patient Characteristics, PML-NBs and SATB1 in Relation to HLA Class I Expression in EBV+ cHL

To investigate the possibility that the effects of PML-NBs and SATB1 expression depend on each other or on other affecters of HLA class I expression, we performed a multivariable analysis. Because the percentage of SATB1+ HRS cells and the SATB1 staining intensity are mutually dependent, we only fitted the percentage of SATB1+ HRS cells into the model. The number of PML-NBs was the most significant independent factor correlating with HLA class I staining (p = 0.008), whereas the percentage of SATB1 also proved to be an independent variable (p = 0.024, Table 1). Age and cHL subtype were no independent contributors to HLA class I expression.

## Discussion

The mechanism of enhanced expression of HLA class I in EBV+ cHL, as well as the down regulation of HLA class I in part of EBV+ and EBV− cHL is unclear. In this study, we showed that the enhanced HLA class I expression in EBV+ cHL is positively associated with the number of PML-NBs and inversely correlated with the nuclear staining intensity and the percentage of SATB1 positive tumor cells. There was no association between HLA class I negative cHL cases and either PML or SATB1, indicating that other mechanisms contribute to down regulation of HLA class I expression, e.g. loss of the HLA loci, aberrant transcription factor expression or epigenetic modifications.

In our case series, retention of HLA class I expression was observed in ∼20% of EBV− and ∼75% of EBV+ cHL. An even stronger than normal HLA class I expression was seen in half of the HLA class I positive EBV+ cHL cases. It is enigmatic how tumor cells benefit from retained or enhanced HLA class I expression in the face of anti-EBV immune responses. It is known that during primary lytic EBV infection of a naïve B cell the HLA class I antigen presentation pathway is strongly inhibited by lytic EBV proteins (BNLF2a, BNLF5 and BILF1) [20], [21], [22]. However, when lytic infection switches to latent infection, the expression of HLA class I is restored. Moreover, it has been reported that LMP1 can enhance HLA class I expression levels to higher than normal [23]. In EBV+ cHL with high HLA class I expression there is an increased number of CTLs in the reactive infiltrate, suggesting that antigens are presented in the context of HLA class I. However, these CTLs are usually not in the close vicinity of HRS cells [6]. Previous studies from our group showed a strong association of two HLA-A types with EBV+ cHL [24], [25]. HLA-A*01 is a risk type and has low affinity for LMP1 and LMP2 derived antigenic peptides, whereas HLA-A*02 is a protective type that has a relatively high affinity for LMP1 and LMP2 derived antigenic peptides [26], [27]. Thus, lack of the HLA-A*02 type might explain why EBV+ HRS cells with HLA class I expression are not recognized and targeted by cytotoxic T cell mediated antitumor responses. In addition, HRS cells express immunosuppressive cytokines, such as TGF-β and IL-10 which can inhibit the cytotoxicity of these CTLs [28], [29]. A possible advantage of HLA class I expression would be inhibition of natural killer (NK) cells. We have previously shown that HLA class I negative HRS cells commonly express the NK cell inhibiting cell surface protein HLA-G instead [5]. Whether EBV+ HRS cells would benefit from an even increased HLA class I expression and what mechanism(s) would be involved remain open questions.

Enhanced HLA class I expression was correlated with an increased number of PML-NBs in EBV+ HRS cells. This increase was not due to an increase in size of HRS cell nuclei. In normal B and T lymphocytes the number of PML-NBs ranged from 3 to 5 per nucleus. We estimate that in HRS cells the number of PML-NBs ranges from close to zero to up to 170 PML-NBs per nucleus. The positive correlation of the number of PML-NBs with HLA class I expression is consistent with the results of a previous study showing that inhibition of PML-III and PML-V isoforms resulted in a down regulation of the expression of a distinct set of HLA class I genes in Jurkat cells [14]. Overexpression of LMP1 in lung epithelial cells induced PML protein expression and increased both the size and number of PML-NBs [30]. Considering that LMP1 can increase HLA class I in Burkitt lymphoma [23], we propose that this LMP1-mediated increase of HLA class I expression might be induced via induction of the number of PML-NBs.

We showed an inverse correlation between SATB1 and enhanced HLA class I expression in EBV+ cHL cases. This is consistent with previous findings in Jurkat cells showing that silencing of SATB1 increases HLA class I expression at the mRNA level from normal to strong [14]. Low SATB1 expression in EBV+ cHL was observed previously by microarray profiling of micro-dissected HRS cells [31]. Of note, SATB1 staining was more variable in HLA class I negative cases and not stronger in comparison to cases with normal expression. Therefore, there is no evidence in our study that increased SATB1 expression is associated with down regulation of HLA class I. We conclude that lack of SATB1 is only associated with stronger than normal HLA class I staining in EBV+ cHL.

Since SATB1 partially resides in PML-NBs we were interested to see whether these two factors have an independent effect on HLA class I expression. Multivariable analysis indicated that the number of PML-NBs is the most significant independent factor correlating with HLA class I expression in EBV+ cHL, while the percentage of SATB1+ tumor cells also had an independent effect. Although sometimes a subtle dot-like nuclear SATB1 staining could be observed, diffuse SATB1 nuclear staining prohibited scoring of SATB1 containing PML-NB. It is therefore unclear if the effect of SATB1 is caused by SATB1 located in PML-NBs, by diffuse SATB1 or by both.

In conclusion, strongly enhanced HLA class I expression in EBV+ cHL is positively correlated with the number of PML-NBs and negatively correlated with SATB1 at the protein level. SATB1 and PML may play an important role in up regulating HLA class I expression in EBV+ cHL.

## Supporting Information

Table S1 **Antibodies used for immunohistochemistry.**
(DOC)

Table S2 **Patient characteristics, HLA class I, SATB1 and PML-NBs in EBV+ and EBV− cHL.**
(DOC)

Table S3 **Clinical and pathologic characteristics and HLA class I, SATB1 and PML staining results for all cHL patients.**
(DOC)
