# Supplementary material for: PML Nuclear Bodies and SATB1 Are Associated with HLA Class I Expression in EBV+ Hodgkin Lymphoma
Source: PLoS One. 2013 Aug 29;8(8):e72930. doi: 10.1371/journal.pone.0072930 (PMC3757028; doi:10.1371/journal.pone.0072930)
Supplement: Table S1 — Antibodies used for immunohistochemistry. (DOC) [file pone.0072930.s001.doc]

Table S1. Antibodies used for immunohistochemistry.

| **Primary antibody** | **Clone** | **Supplied by** | **Antigen retrieval method^a^** | **Dilution** | **Incubation** |
| --- | --- | --- | --- | --- | --- |
| Mouse anti-human HLA class I heavy chain antibody^b^ | HC10 | Prof. dr. J. Neefjes^c^ | 95°C, 15 min in microwave oven | 1:200 | RT, 1 hour |
| Rabbit anti-human β2-microglobulin antibody | polyclonal | DAKO | 95°C, 15 min in microwave oven | 1:200 | RT, 1 hour |
| Mouse anti-human PML antibody | PG-M3 | Santa Cruz Biotechnology | 125°C, 15 min in pressure cooker | 1:3200 | 4°C, overnight |
| Mouse anti-human SATB1 antibody | 14/SATB1 | BD Biosciences | 125°C, 15 min in pressure cooker | 1:3200 | 4°C, overnight |

^a^ Antigen retrieval buffer is Tris-EDTA (pH 9.0) for all the antibodies; ^b^ The HC10 antibody detects HLA-B and HLA-C and a few infrequent alleles of HLA-A; ^c^ the Netherlands Cancer Institute, Amsterdam

RT, room temperature
