# Supplementary material for: PML Nuclear Bodies and SATB1 Are Associated with HLA Class I Expression in EBV+ Hodgkin Lymphoma
Source: PLoS One. 2013 Aug 29;8(8):e72930. doi: 10.1371/journal.pone.0072930 (PMC3757028; doi:10.1371/journal.pone.0072930)
Supplement: Table S2 — Patient characteristics, HLA class I, SATB1 and PML-NBs in EBV+ and EBV− cHL. (DOC) [file pone.0072930.s002.doc]

Table S2. Patient characteristics, HLA class I, SATB1 and PML-NBs in EBV+ and EBV- cHL.

|  | EBV status | | | | p value |
| --- | --- | --- | --- | --- | --- |
|  | negative (n=27) | | positive (n=54) | |  |
|  | n | % | n | % |  |
| **Age, median** |  |  |  |  |  |
| median | 25 | | 34 | | 0.01^a^ |
| (min-max) | (9-88) | | (7-94) | |  |
| **Age, years** |  |  |  |  |  |
| 0-14 | 5 | 18.5 | 9 | 16.7 |  |
| 15-44 | 21 | 77.8 | 17 | 31.5 | <0.001^b^ |
| 45-64 | 0 | 0.0 | 16 | 29.6 |  |
| >=65 | 1 | 3.7 | 12 | 22.2 |  |
| **Gender** |  |  |  |  |  |
| M | 12 | 44.4 | 33 | 61.1 | 0.17^c^ |
| F | 15 | 55.6 | 21 | 38.9 |  |
| **Clinical stage** |  |  |  |  |  |
| I | 3 | 11.1 | 14 | 25.9 |  |
| II | 14 | 51.9 | 17 | 31.5 | 0.19^b^ |
| III | 5 | 18.5 | 13 | 24.1 |  |
| IV | 1 | 3.7 | 1 | 1.9 |  |
| unknown | 4 | 14.8 | 9 | 16.7 |  |
| **Hodgkin subtype** |  |  |  |  |  |
| Nodular Sclerosis | 26 | 96.3 | 35 | 64.8 | <0.01^b^ |
| Mixed Cellularity | 0 | 0.0 | 10 | 18.5 |  |
| LR/LD/NOS | 1 | 3.7 | 9 | 16.7 |  |
| **HLA class I staining intensity** |  |  |  |  |  |
| negative | 22 | 81.5 | 14 | 25.9 |  |
| normal | 5 | 18.5 | 19 | 35.2 | <0.0001^b^ |
| strong | 0 | 0.0 | 21 | 38.9 |  |

^a^ Mann-Whitney test; ^b^ Fisher’s exact test; ^c^ Chi-square test

LR, lymphocyte rich. LD, lymphocyte deleted. NOS, not otherwise specified.
