# Supplementary material for: PML Nuclear Bodies and SATB1 Are Associated with HLA Class I Expression in EBV+ Hodgkin Lymphoma
Source: PLoS One. 2013 Aug 29;8(8):e72930. doi: 10.1371/journal.pone.0072930 (PMC3757028; doi:10.1371/journal.pone.0072930)
Supplement: Table S3 — Clinical and pathologic characteristics and HLA class I, SATB1 and PML staining results for all cHL patients. (DOC) [file pone.0072930.s003.doc]

Table S3. Clinical and pathologic characteristics and HLA class I, SATB1 and PML staining results for all cHL patients.

| **case number** | **age at diagnosis** | **sex** | **EBV status** | **subtype** | **stage** | **HC10 intensity** | **SATB1 %** | **SATB1 intensity** | **PML-NBs** |
| --- | --- | --- | --- | --- | --- | --- | --- | --- | --- |
| 1 | 60 | F | + | NS | 3 | - | 0 | - | ≤10 |
| 2 | 23 | M | + | NS | 2 | - | 0 | - | ≤10 |
| 3 | 22 | M | + | NS | 3 | - | 30 | - | ≤10 |
| 4 | 31 | F | + | NS | 2 | - | 40 | - | ≤10 |
| 5 | 8 | M | + | NS | 2 | - | 40 | - | ≤10 |
| 6 | 45 | F | + | NS | 3 | - | 40 | - | ≤10 |
| 7 | 13 | M | + | NS | 3 | - | 60 | - | ≤10 |
| 8 | 22 | M | + | NS | 2 | - | 100 | - | ≤10 |
| 9 | 72 | F | + | NS | 3 | - | 70 | + | ≤10 |
| 10 | 17 | F | + | NS | 2 | - | 90 | + | ≤10 |
| 11 | 59 | F | + | NS | 1 | - | 100 | + | ≤10 |
| 12 | 17 | F | + | NS | 2 | - | 100 | + | ≤10 |
| 13 | 13 | F | + | NS | 2 | - | 100 | + | ≤10 |
| 14 | 25 | M | + | NS | 3 | - | 100 | + | >10 |
| 15 | 49 | M | + | LR | 2 | + | 0 | - | ≤10 |
| 16 | 13 | M | + | MC | 2 | + | 0 | - | ≤10 |
| 17 | 37 | F | + | NS | 3 | + | 0 | - | ≤10 |
| 18 | 43 | M | + | NS | 1 | + | 0 | - | ≤10 |
| 19 | 8 | M | + | NS | 1 | + | 50 | + | ≤10 |
| 20 | 49 | M | + | MC | ? | + | 80 | + | >10 |
| 21 | 42 | M | + | NS | 2 | + | 80 | + | ≤10 |
| 22 | 67 | F | + | NOS | 3 | + | 90 | + | ≤10 |
| 23 | 60 | M | + | NOS | 1 | + | 100 | + | ≤10 |
| 24 | 13 | F | + | NS | 2 | + | 100 | + | >10 |
| 25 | 88 | M | + | LD | ? | + | 100 | + | ≤10 |
| 26 | 77 | F | + | LD | ? | + | 100 | + | ≤10 |
| 27 | 42 | M | + | MC | 1 | + | 100 | + | ≤10 |
| 28 | 46 | M | + | NS | ? | + | 100 | + | ≤10 |
| 29 | 50 | F | + | MC | 4 | + | 90 | + | ≤10 |
| 30 | 83 | F | + | NS | 1 | + | 100 | + | >10 |
| 31 | 77 | F | + | NOS | ? | + | 100 | + | ≤10 |
| 32 | 27 | M | + | MC | 3 | + | 100 | + | ≤10 |
| 33 | 53 | M | + | NS | 2 | + | 100 | + | ≤10 |
| 34 | 20 | M | + | NS | 1 | ++ | 0 | - | >10 |
| 35 | 67 | F | + | MC | 3 | ++ | 0 | - | ≤10 |
| 36 | 62 | M | + | NS | ? | ++ | 0 | - | ≤10 |
| 37 | 32 | F | + | NS | 1 | ++ | 0 | - | ≤10 |
| 38 | N.D | F | + | MC | 1 | ++ | 0 | - | >10 |
| 39 | 60 | M | + | NS | 1 | ++ | 0 | - | ≤10 |
| 40 | 15 | M | + | NS | 1 | ++ | 0 | - | ≤10 |
| 41 | 70 | M | + | NS | ? | ++ | 0 | - | ≤10 |
| 42 | 57 | M | + | NS | 1 | ++ | 0 | - | >10 |
| 43 | 53 | F | + | NS | ? | ++ | 70 | - | >10 |
| 44 | 63 | F | + | NOS | 2 | ++ | 70 | - | >10 |
| 45 | 36 | M | + | NS | 2 | ++ | 80 | - | >10 |
| 46 | 7 | F | + | NOS | 3 | ++ | 30 | + | >10 |
| 47 | 69 | M | + | NS | 3 | ++ | 40 | + | >10 |
| 48 | 8 | M | + | NS | 2 | ++ | 60 | + | >10 |
| 49 | 74 | M | + | NS | 2 | ++ | 70 | + | ≤10 |
| 50 | 56 | M | + | NOS | 2 | ++ | 80 | + | >10 |
| 51 | 45 | M | + | MC | 3 | ++ | 100 | + | >10 |
| 52 | 71 | M | + | MC | ? | ++ | 100 | + | >10 |
| 53 | 27 | M | + | NS | 1 | ++ | 100 | + | ≤10 |
| 54 | 8 | M | + | MC | 1 | ++ | 100 | + | ≤10 |
| 55 | 17 | M | - | NS | 2 | - | 0 | - | ≤10 |
| 56 | 32 | F | - | NS | ? | - | 0 | - | ≤10 |
| 57 | 43 | M | - | NS | ? | - | 0 | - | ≤10 |
| 58 | 34 | M | - | NS | 1 | - | 10 | - | ≤10 |
| 59 | 18 | F | - | NS | 2 | - | 10 | - | ≤10 |
| 60 | 13 | F | - | NS | 2 | - | 20 | - | >10 |
| 61 | 22 | F | - | NS | 2 | - | 70 | - | ≤10 |
| 62 | 37 | F | - | NS | 2 | - | 40 | + | >10 |
| 63 | 13 | F | - | NS | 3 | - | 70 | + | >10 |
| 64 | 30 | M | - | NS | 3 | - | 70 | + | ≤10 |
| 65 | 31 | M | - | NS | 2 | - | 80 | + | ≤10 |
| 66 | 19 | F | - | NS | 2 | - | 80 | + | ≤10 |
| 67 | 14 | F | - | NS | 2 | - | 90 | + | ≤10 |
| 68 | 20 | F | - | NS | 3 | - | 90 | + | ≤10 |
| 69 | 20 | F | - | NS | 2 | - | 90 | + | ≤10 |
| 70 | 14 | F | - | NS | 1 | - | 90 | + | ≤10 |
| 71 | 28 | M | - | NS | 3 | - | 90 | + | >10 |
| 72 | 9 | M | - | NS | 2 | - | 100 | + | ≤10 |
| 73 | 25 | M | - | NS | 4 | - | 100 | + | ≤10 |
| 74 | 40 | F | - | NS | 2 | - | 100 | + | >10 |
| 75 | 18 | M | - | NS | 2 | - | 100 | + | ≤10 |
| 76 | 30 | F | - | NS | 2 | - | 100 | + | ≤10 |
| 77 | 36 | M | - | NS | 2 | + | 0 | - | ≤10 |
| 78 | 42 | M | - | NS | ? | + | 10 | - | >10 |
| 79 | 19 | F | - | NS | 3 | + | 30 | + | ≤10 |
| 80 | 88 | M | - | LR | ? | + | 80 | + | ≤10 |
| 81 | 40 | F | - | NS | 1 | + | 90 | + | ≤10 |

NS, nodular sclerosis. MC, mixed cellularity. LR, lymphocyte rich. LD, lymphocyte deleted. NOS, not otherwise specified.
